# The relationship between psychosocial distress and oral health status in patients with facial burns and mediation by oral health behaviour

**DOI:** 10.1186/s12903-021-01532-0

**Published:** 2021-04-01

**Authors:** Farooq Ahmad Chaudhary, Basaruddin Ahmad

**Affiliations:** 1 School of Dentistry, Shaheed Zulfiqar Ali Bhutto Medical University, Islamabad, Pakistan; 2 School of Dental Sciences, Universiti Sains Malaysia, Kubang Kerian, Malaysia

**Keywords:** Burn, Oral health, Psychological, Mediation, Disfigurement, Behaviours

## Abstract

**Background:**

There is limited discussion on the influence of psychosocial factors on the oral health of patients with a facial burn injury. This report investigated the relationship between oral health and psychosocial distress in patients with facial burns and the role of oral health behaviour in mediating the relationship.

**Methods:**

The data were part of a cross-sectional study that had systematically and randomly selected patients with > 10% total burn surface area from a burn centre in Pakistan. The oral health status (DMFT, CPI, OHI-S) and severity of facial disfigurement were assessed. Validated instruments in the Urdu language were self-administered and information relating to oral health behaviour (brushing and dental visits), oral health-related quality of life (OHIP-14), satisfaction with appearance, self-esteem, anxiety and depression, resilience, and social support were collected. The statistical analyses included simple linear regression, Pearson correlation, t-test, and ANOVA. Mediation analysis was carried out to examine the indirect effect by oral health behaviour.

**Results:**

From a total of 271 participants, the majority had moderate to severe facial disfigurement (89%), low self-esteem (74.5%), and moderate to high levels of social support (95%). The level of satisfaction with appearance was low, whereas anxiety and depression were high. Disfigurement and satisfaction with appearance were associated with lower self-esteem and social support (*p* < 0.05). Greater severity of disfigurement, higher levels of anxiety and dissatisfaction with appearance, and lower levels of self-esteem and social support were associated with greater DMFT and OHIP-14 scores, worse periodontal and oral hygiene conditions, and less frequent tooth brushing and dental visits (*p* < 0.05). The main barriers to oral healthcare utilization were psychological and social issues (*p* < 0.05). The indirect effect by oral health behaviour was not significant for anxiety but was significant for disfigurement, satisfaction with appearance, self-esteem, and social support.

**Conclusion:**

There is an association between the psychosocial factors and oral health of patients with facial burns through a direct effect and mediation by oral health behaviour.

## Introduction

Burn injury is a traumatic experience that leaves a victim with acute and chronic physical and psychological conditions [1, 2]. The long-term physical complications include deformities, immobility and functional impairments of the affected area, and pain [1, 3]. Post-traumatic psychological complications such as distress, depression, and anxiety [4, 5] also affect health, function, and quality of life in patients with burn injuries [6].

In burn injuries involving the facial area, long-term complications may include effects on oral health. When a burn injury involves the lips and mouth, scar contracture may lead to the distortion of the lips, microstomia, and narrowing of the mouth opening [7]. There may also be discomfort and pain as the scar stretches during oral functions as well as reduced sensation and muscle control around the affected area. The combination of these factors can greatly impair daily activities such as speaking, eating, swallowing, and accessing the oral cavity. The latter impairment can impact oral hygiene care as teeth cleaning becomes less comfortable and inefficient, thus increasing the risk of plaque accumulation and dental diseases. Further complications may include mouth sores due to drooling, teeth grinding, malocclusion, and temporomandibular joint disorder due to muscle incoordination [7–9].

Facial disfigurement also affects social interactions due to the unsightly appearance, difficulty in reading facial expressions, and unclear speech. This can cause psychological distress such as low self-esteem, anxiety, and depression [10]. Evidence has also linked these conditions to less frequent tooth brushing and fewer dental visits [11]. Poor oral health outcomes in individuals with facial burns have been linked to dental anxiety [12]. However, there is little discussion on the influence of psychosocial factors on oral health in these patients. Thus, the objective of the current report was to examine the relationship between psychosocial distress and oral health measures in patients with facial burns. In addition, the study assessed whether oral health behaviours mediated the above-mentioned relationships. There is a need to understand the conditions and mechanisms that affect the oral health of burn victims in order to develop intervention programs to rehabilitate and reintegrate them into society.

## Materials and methods

The current report is part of a cross-sectional study that investigated the oral health status of patients with facial burns at the Burn Care Center of Pakistan Institute of Medical Sciences, Islamabad, Pakistan. Apart from the psychosocial measures, which have not been reported before, the parameters used in the present report have been described earlier in Chaudhary et al. (2019) [12]. The study protocol was reviewed and approved by the ethics committee of the institution (Reference no. F.1-1/2015/ERB/SZABMU). Systematic random sampling was used by selecting every second patient who attended the centre for follow-up. Patients with head and neck burns involving more than 10% of total body surface area who were able to eat by mouth were included. An extra-oral examination was carried out to assess the severity of disfigurement using a single item observer-rated disfigurement scale [13]. The scale ranges from 1 to 9 points and categorized as the minimum (1–3 points), moderate (4–6 points) and severe (7–9 points) disfigurement, according to their score on this scale. An intra-oral examination was by one qualified dentist to assess oral health status according to the World Health Organisation oral health survey methods, including the DMFT, Community Periodontal Index (CPI), and Oral Hygiene Index-Simplified (OHI-S) [14, 15].

The participants completed self-administered, reliable, and validated instruments in the Urdu language to assess their oral health behaviours and psychosocial measures. The oral health behaviour measures included the frequencies of daily tooth brushing (once, twice, or more) and dental check-up in the past year (Yes, No) [16]. The barriers to utilization of oral health care services were assessed using an open-ended question: “*Is there anything, such as cost, anxiety, location, illness, or other problems, that has kept you from going to the dentist?*”. Based on participants’ responses their main reason for not visiting a dentist was categorised as dental anxiety, social, distance, cost, or self-perceived. If participants listed multiple reasons then their first reason was classed as the main reason. [12, 17]. The *Oral Health Impact Profile (OHIP-14)* assesses oral health-related quality of life using 14 items measured on a 5-point Likert scale from “never” (0) to “very often” (4). The total score ranges from 0 to 56, and a lower score indicates a better oral health-related quality of life [18, 19]. *The Satisfaction With Appearance Scale (SWAP)* is a 14-item instrument to measure the self-perceived satisfaction with appearance and socio-behavioural impact of burn scars. Participants rate each item on a 7-point Likert scale from 1 (strongly disagree) to 7 (strongly agree); a higher total score (range: 0–84) indicates a greater dissatisfaction with the facial image [5, 20]. *The Hospital Anxiety and Depression Scale (HADS)* assesses anxiety (7 items) and depression (7 items) on a scale of 0 (less frequently or equivalent) to 3 (more frequently or equivalent). The total score for each psychological condition ranges from 0 to 21, where a higher score indicates a worse condition. For each condition, a score greater than 7 suggests the presence of psychological morbidity [21, 22]. *The Rosenberg Self-Esteem Scale (RSES)* is a 10-item instrument to assess self-worth and self-acceptance on a four-point scale (0–3) ranging from strongly agree to strongly disagree. The total score ranges from 0 to 30 and higher scores indicate a higher level of self-esteem; patients with a total score < 15 are considered as having low self-esteem [23, 24]. *The Multidimensional Scale of Perceived Social Support (MSPSS)* is a 12-item instrument to assess perceived social support using a 7-point Likert scale which ranges very strongly disagree (1) to very strongly agree (7). The total score is divided by 12 to give a mean score that ranges from 1 to 7, where a greater score corresponds to better perceived social support, and re-categorised as low (score: 1–2.9), moderate (3–5) and high (5.1–7) levels of support [25, 26]. *The Brief Resilience Scale (BRS)* assesses resilience using a 6-item instrument with 5-point Likert scale responses ranging from “strongly disagree” (1) to “strongly agree” (5). The total score of all items is divided by 6 to give a mean score that indicates low (1) to high (5) resilience [27, 28]. All data collection was carried out by FAC, who also underwent training from the burn specialists at the centre to measure disfigurement in a clinical setting.

### Statistical analyses

Summary statistics were obtained for all the variables. Bivariate association analyses between the psychosocial factors and oral health outcomes and behaviours were conducted using Pearson correlation, t-test, ANOVA with post hoc tests and simple linear regression. Mediation analysis was carried out to examine the hypothesis that oral health behaviours mediate the influence of psychological factors on oral health. Instead of using the original form, the mediator and oral health outcome variables were aggregated using principal component analysis (PCA) without rotation to simplify the analysis and interpretation. The clinical and oral health-related quality of life measures were combined and re-defined as *oral health outcome* (Eigenvalue 3.30, 82.5% of variance explained), in which a larger value indicates a worse oral health condition. Tooth brushing and dental visits were combined to form *health behaviour* (Eigenvalue 1.57, 78.5% of variance explained); a larger value indicates better health behaviour. For both aggregated parameters, the mean = 0 and SD = 1. Analyses were carried out to examine the indirect effect of each psychosocial factor (stressor) on oral health outcome through health behaviour (psychosocial stressor → health behaviour → oral health outcome) based on Model 4 of the PROCESS macro v3.4.1 [29]. The assumptions and diagnostic of the statistical methods were performed for all analysis. For normality assumption, the graphical method showed that the variables were approximately normally distributed with the skewness and kurtosis less than ± 2. The significance level was set at 5% and analysis was conducted using IBM SPSS v26.0.

## Results

A total of 300 patients were invited to participate in the study, 20 had declined and 9 incomplete responses were omitted; only N = 271 (90.3%) were available for analysis. The sample characteristics and oral health status were described in an earlier report [12]. In summary, the majority of the sample were females (68.6%), under 35 years old (78.9%), unemployed (49.1%), and from the low-income group (65.7%), and had 6–12 years of schooling (64.9%). The mean DMFT and overall OHIP-14 scores were 11.0 (SD = 2.4) and 37.7 (SD = 8.5) respectively. Most participants had periodontal pockets ≥ 4 mm in at least one site (59%), poor oral hygiene (66.1%), practised tooth brushing once a day (78%), and did not visit a dentist in the past year for a regular check-up (89%). Participants most commonly cited anxiety-related issues as the most important barrier to utilising oral health care services (46% of participants), followed by the cost of treatment (25%) and social issues (15.5%).

The summary statistics of the psychosocial measures are presented in Table 1. The majority of the participants had moderate to severe facial disfigurement (89%), low self-esteem (74.5%), and moderate to high levels of social support (95%). The high mean scores suggested high levels of anxiety, depression, and dissatisfaction with the appearance among the participants. Correlation analysis showed that the severity of disfigurement was positively and strongly correlated with dissatisfaction with appearance mildly with depression. The severity of disfigurement and satisfaction with appearance were both negatively and moderately correlated with self-esteem and social support (*p* < 0.05). Positive and moderate correlations were also found between anxiety and depression and self-esteem and social support (*p* < 0.05).

**Table 1:** Distribution and summary statistics of the psychosocial measures (n = 271)

Psychological instruments	Number (%)	Mean (SD)
*Disfigurement*		6.21 (1.5)
Minimum	30 (11.1)	2.97 (0.18)
Moderate	114 (42.1)	5.73 (0.63)
Severe	127 (46.9)	7.42 (0.61)
Satisfaction with appearance scale (SWAP)	–	69.69 (7.1)
*Hospital anxiety and depression scale (HADS)*		
Anxiety	–	13.77 (3.2)
Depression	–	14.24 (4.3)
*Rosenberg self-esteem scale (RSES)*		13.21 (1.89)
Normal self-esteem	68 (25.1)	15.81 (1.03)
Low self-esteem	203 (74.9)	12.32 (1.17)
*Multidimensional scale of perceived social support (MSPSS), level of support*		4.83 (0.8)
Low	12 (4.4)	2.64 (0.23
Moderate	123 (45.4)	4.33 (0.46)
High	136 (50.2)	5.48 (0.24)
Brief resilience scale (BRS)	–	2.97 (0.2)

The analysis showed significant associations between psychosocial factors and oral health. Increased severity of disfigurement, SWAP, and anxiety was associated with greater DMFT and OHIP-14 (*p* < 0.05); and positively correlated with the CPI and OHI-S indices (*p* < 0.05) (Table 2). Better self-esteem and social support (greater scores) were associated with lower DMFT and OHIP-14 scores (*p* < 0.001) and negatively and moderately correlated with the CPI and OHI-S indices (*p* < 0.001). The mean scores for disfigurement and SWAP were greater in those who brush the teeth and visit the dentist less frequently (*p* < 0.001) and, correspondingly, the mean scores were lower for self-esteem and social support (*p* < 0.001) (Table 3). In participants with dental anxiety, the mean scores of disfigurement, SWAP, depression, self-esteem, and social support were significantly different from at least one other barrier to oral health care use (Table 3). Similarly, in those with social barriers, the mean scores of depression and self-esteem were different from the distance and self-perceived barriers.

**Table 2:** Associations between oral health outcomes and the psychosocial measures

Psychosocial	DMFT		CPI Mean (SD)					OHI-S Mean (SD)				OHIP-14 Mean (SD)	
	Regression coefficient (95% CI)	p	Bleeding n = 31	Calculus n = 80	Pocket 4–5 mm n = 117	Pocket ≥ 6 mm n = 43	r	Good n = 7	Fair n = 85	Poor n = 179	r	Regression coefficient (95% CI)	p
Disfigurement	1.38 (1.29, 1.48)	< 0.001	3.5 (0.9)	5.6 (1.1)	6.8 (0.7)	7.4 (1.0)	0.74^1^	2.8 (0.3)	5.0 (1.4)	6.9 (0.9)	0.68^1^	4.57 (4.18, 4.96)	< 0.001
SWAP	0.27 (0.24, 0.28)	< 0.001	52.8 (5.7)	62.7 (6.1)	68.7 (4.2)	71.5 (4.8)	0.70^1^	49.5 (3.7)	59. (7.3)	68.9 (4.7)	0.66^1^	0.85 (0.75, 0.93)	< 0.001
HADS													
Anxiety	0.09 (0.001, 0.17)	0.04	12.9 (3.2)	13.0 (3.4)	14.3 (3.2)	14.0 (2.5)	0.15^1^	11.5 (3.5)	13.4 (3.1)	14.0 (3.2)	0.12^2^	0.34 (0.02, 0.64)	0.03
Depression	0.02 (− 0.04, 0.08)	0.49	14.2 (4.4)	13.3 (4.2)	14.6 (4.4)	14.8 (4.0)	0.09	12.0 (5.0)	14.0 (3.7)	14.4 (4.5)	0.07	0.09 (− 0.14, 0.33)	0.40
RSES	− 0.69 (− 0.82, − 0.56)	< 0.001	15.5 (1.5)	13.6 (1.8)	12.5 (1.5)	12.4 (1.2)	− 0.47^1^	16.0 (0.5)	14.0 (1.9)	12.6 (1.6)	− 0.42^1^	− 2.43 (− 2.89, − 1.98)	< 0.001
MSPSS	− 2.0 (− 2.26, − 1.74)	< 0.001	5.5 (0.4)	5.1 (0.5)	4.7 (0.6)	3.9 (0.9)	− 0.57^1^	5.8 (0.1)	5.3 (0.5)	4.5 (0.8)	− 0.46^1^	− 5.52 (− 6.58,—4.46)	< 0.001
BRS	0.03 (− 1.59, − 1.65)	0.97	2.9 (0.1)	2.9 (0.1)	2.9 (0.1)	2.9 (0.1)	− 0.001	2.9 (0.1)	2.9 (0.1)	2.9 (0.1)	0.31	− 1.32 (− 7.05, 4.41)	0.65

r: Pearson correlation

^1^*p* < 0.001; ^2^*p* < 0.05

**Table 3:** Associations between oral health behaviors and the psychosocial measures

Psychosocial	Tooth brushing			Dental visits			Barriers to oral health care					
	Once N = 212 Mean (SD)	Twice or more N = 59 Mean (SD)	p^t^	No N = 242 Mean (SD)	Yes N = 29 Mean (SD)	p^t^	Dental anxiety N = 125 Mean (SD)	Social N = 42 Mean (SD)	Distance N = 25 Mean (SD)	Cost N = 68 Mean (SD)	Self-perceived N = 11 Mean (SD)	p^a^
Disfigurement	6.6 (1.1)	4.5 (1.5)	< 0.001	6.4 (1.2)	4.0 (1.4)	< 0.001	6.7 (1.1)^A^	6.1 (1.5)	6.0 (1.4)	5.7 (1.7)	4.6 (1.9)	< 0.001^1^
SWAP	67.7 (5.7)	57.6 (7.9)	< 0.001	66.9 (6.3)	54.5 (7.5)	< 0.001	67.7 (5.9)^E^	65.1 (7.5)	64.6 (7.4)	63.5 (8.4)	58.5 (10.4)	< 0.001^1^
HADS												
Anxiety	13.9 (3.1)	13.1 (3.4)	0.86	13.8 (3.2)	13.4 (2.8)	0.52	13.9 (2.8)	14.1 (3.3)	13.4 (3.4)	13.8 (3.5)	11.2 (3.2)	0.81
Depression	14.0 (4.0)	13.6 (4.0)	0.27	14.2 (4.4)	14.3 (3.8)	0.85	14.1 (3.9) ^B^	15.4 (4.7)^C^	12.0 (4.5)^D^	15.0 (4.5)	12.3 (3.4)	0.007^2^
RSES	12.8 (1.6)	14.4 (2.1)	< 0.001	12.9 (1.7)	15.2 (2.0)	< 0.001	12.9 (1.7)^F^	12.9 (1.4)^G^	13.7 (1.9)	13.6 (2.1)	14.6 (1.8)	0.003^2^
MSPSS	4.6 (0.8)	5.4 (0.5)	< 0.001	4.7 (0.8)	5.6 (0.2)	< 0.001	4.7 (0.7)	4.8 (0.9)	4.7 (0.8)	4.9 (0.8)	5.4 (0.6)	0.99
BRS	2.9 (0.1)	2.9 (0.1)	0.24	2.9 (0.1)	2.9 (0.1)	0.73	2.9 (0.1)	3.0 (0.2)	3.0 (0.2)	3.0 (0.1)	3.0 (0.1)	0.31

t: t-test; a: ANOVA

Posthoc: ^1^Games-Howell, ^2^LSD

Significant pairwise (*p* < 0.05): A vs Cost, Self-Perceived; B vs Distance; C vs Distance, Self-perceived; D vs Cost; E vs Cost; F vs Cost, Distance, Self-Perceived; G vs Self-Perceived

Resilience and depression were excluded from the mediation analysis because they were not related to oral health outcomes. The mediation analysis showed significant indirect effects of disfigurement, SWAP, self-esteem, and social support on oral health outcome, where the mediation by health behaviour explained 18%, 23%, 41%, and 34% of the relationship between the psychosocial factors and oral health outcome respectively (see Table 4). The indirect effect of anxiety through health behaviour was not significant.

**Table 4:** Results of the mediation analysis to examine the indirect effect of the psychosocial factors on oral health outcome

Stressor	Stressor → OH coefficient (se)	Stressor → HB coefficient (se)	HB → OH coefficient (se)	Indirect effect coefficient (Bootstrap 95% CI)	Indirect effect (%)
Disfigurement	0.57 (0.021)^2^	− 0.41 (0.032)^2^	− 0.25 (0.036)^2^	0.10 (0.067, 0.146)	18.1
SWAP	0.11 (0.005)^2^	− 0.08 (0.006)^2^	− 0.31 (0.04)^2^	0.02 (0.016, 0.034)	23.1
Anxiety	0.05 (0.019)^1^	− 0.03 (0.019)	− 0.68 (0.044)^2^	0.02 (− 0.060, 0.043)	–
RSES	− 0.29 (0.027)^2^	0.22 (0.030)^2^	− 0.56 (0.045)^2^	− 0.12 (− 0.171, − 0.077)	41.4
MSPSS	− 0.76 (0.058)^2^	0.50 (0.068)^2^	− 0.52 (0.042)^2^	− 0.26 (− 0.360, − 0.181)	34.2

HB: Oral health behaviour, OH: Oral health

^1^< 0.05, ^2^< 0.001

## Discussion

The study had examined the relationship between psychosocial factors and oral health in patients with facial burns and whether oral health behaviours mediate the relationship. The results showed that poor oral health conditions and oral health-related quality of life are associated with greater severity of disfigurement, dissatisfaction with appearance and anxiety, and lower self-esteem and social support. It also showed an association between poor psychosocial status and oral health behaviour. These findings are consistent with previous reports in that adverse psychological status is associated with a greater risk of developing oral diseases [30, 31]. Nevertheless, the mechanism that explains the relationship is not as clear and direct as that for the influence of oral health behaviour, where poor personal and professional oral health care increases plaque accumulation and the risk of common oral diseases [32, 33]. Except for depression, which is claimed to reduce the immune response in the development of periodontal disease [30], there is little discussion in the literature to explain a direct involvement of anxiety, dissatisfaction with appearance, self-esteem, and social support in the development of caries and periodontal diseases. It is more rational to assume that psychosocial factors influence oral health through oral behaviour practices. Following that hypothesis, this study examined the data for evidence of mediation in the relationship between psychosocial factors and oral health outcomes. The results showed the indirect effect of anxiety was not significant, but there were significant indirect effects of disfigurement, dissatisfaction with appearance, self-esteem, and social support on oral health status, mediated by oral health behaviour. Only one other study investigated the role of oral health behaviour as a mediator but the effect of maternal education level at birth on gingival bleeding was insignificant [34]. Socio-demographic factors such as age, gender, and income had been considered and examined in the mediation analysis but they did not meet the assumption of the analysis and thus, excluded from the current report.

Besides the statistical evidence, there are also other rationales for the mediation model. As previously mentioned, physical changes such as those caused by deformity to the facial region can physically influence oral health practice. Many participants in the current study were observed to have severe facial and lip deformities, and experienced pain and discomfort during mouth opening. This may influence the attitude towards, and practice of, personal oral hygiene care. However, issues such as the extent to which disfigurement affects brushing efficiency, oral hygiene care practice, and/or adequacy of oral health literacy and skills of the participants are not clear from the present study and require further investigation.

The deformity may also influence the socio-behaviour of the participants which indirectly affects dental treatment-seeking behaviour. Patients with facial burns are afraid to look at people, cover their faces because they feel apprehensive when they are stared at, avoid social environments, dislike going outdoors, and prefer to stay indoors for fear of societal stigma [35]. However, accessing oral health services requires them to travel and mix in a crowded environment, particularly when using public transportation, and puts them in undesirable and uncomfortable situations [36–38]. To avoid these, some participants may delay or cancel an appointment [39, 40] and lose out on professional help. The current study supports this as some participants listed stigma and embarrassment as a (social) barrier to oral health care utilization.

There was a low level of self-esteem and a moderate level of support among the participants [41]; these factors were inversely associated with oral health outcomes, consistent with earlier literature [42, 43]. Adjusting to life adversities requires more than just innate capabilities such as coping skills, resilience, personality, and an individual will power; strong support from the people close to them is also greatly beneficial [44, 45]. A typical Pakistani family is generally religious, family-oriented, and always willing to assist each other, particularly in matters involving health, finance, and moral issues [46]. The level of support is also dependent on the size of the social network, the closeness of family kinship, and available resources. Because most participants are from less affluent backgrounds, find it difficult to return to work and rely on others for financial and logistical support [47–49], they may also find dental treatment to be costly. Having better support and self-esteem is an advantage as they can buffer the adverse effect of burn injury.

The level of anxiety among the participants was moderate and its effect on oral health was not mediated by oral health behaviour. The anxiety, measured using the HADS, is likely to reflect dental anxiety, which is described as shyness, nervousness, and fear of dental treatment, and confirmed by the participants’ responses to the question on the barrier to utilization of oral health care [12]. Previously, a systematic review found no evidence for the relationship between anxiety and dental caries and periodontal disease but the meta-analysis did, however, report an association between dental anxiety and caries severity, but not with periodontal disease and tooth loss [31].

The high depression levels among the participants are consistent with previous reports but no association with oral health was found in the present study despite considerable evidence, including from systematic reviews [10, 50–52]. This may be caused by the small variation in scores between the participants and/or difficulty to discriminate the comorbidity of anxiety and depression by the participants [31, 53]. Previous evidence linked depression to health-risk behaviours such as increased consumption of carbohydrate-rich meals and snacks and less frequent dental attendance, brushing, and flossing [54–56]. Furthermore, taking antidepressant medication reduces saliva secretion and increases cortisol levels [56–58] which increases the risk of common oral diseases. Another factor, resilience, is reported to be protective against stress and assists in post-burn life adjustment in burn patients [59, 60]. However, it is not associated with oral health outcomes in the present study, possibly because the resilience instrument is not a reliable measure in patients with facial burns.

Interpretation from the findings should also consider the circumstances surrounding the participants, including those not captured in the data. A reflection on the data collection process revealed that most of the participants seemed uncomfortable, hesitant, and shy. They were anxious, emotional, and depressed when asked about the history and implications of the injury. Female participants often hesitated and had to be persuaded to respond to the SWAP and MSPSS instruments and cause of burn injuries; most noticeably in chemical or acid burn victims. Some were reluctant to respond to the questionnaires in the presence of individuals who accompany them to the burn centre and cooperated only after they were separated, which raises questions for the reason behind it. Many of them were also financially dependent on their nonaffluent families. These observations suggest that there is a deeper complex psychosocial issue in this disadvantaged population that requires further attention and investigation. These barriers to care must be lifted successfully [31] before oral health intervention can be pursued. Programs to improve social interaction skills could be implemented. Cognitive–behavioural therapy can help facially disfigured patients to overcome social isolation, stress, and anxiety problems [61, 62]. Programs that train and educate burn victims to effectively anticipate and control their emotions and respond positively to the reactions of others, build self-confidence and esteem and equip patients with the methods and strategies to manage adverse situations are recommended before a patient is discharged from hospital [63]. The availability of such programs is, however, very limited in non-developed countries. Further studies should explore the compatibility of such program with regards to the local cultural and social issues before they are emulated in Pakistan. It is also recommended that burn specialists are made aware of the specific long- and short-term oral health issues in patients with facial burns and refer them to oral health professionals.

Some limitations of this study have been discussed previously, including the inference from the cross-sectional study design, lack of reliability because the patients were unwilling to return for clinical reassessment, and recall bias [12]. Limitations relating to the reliability of the instruments are also addressed above. Interpretation is also limited because patients with facial burns who receive follow-up at an institution are not representative of the general population. Hence, the results should be interpreted with caution. The key strength of this study is in its originality; this is the first study to investigate the impact of psychosocial distress on the oral health of patients with facial burns and highlights a niche and underserved population. It also provides potential evidence for the role of oral health behaviour as a mediator for the effect of psychosocial distress.

## Conclusions

This study shows an association between psychosocial distress and poor oral health status and oral health-related quality of life in facial burn patients; furthermore, it shows this relationship is mediated by oral health behaviours. Patients with facial burns should be trained to resolve their psychosocial issues to help them overcome the barriers to seeking professional dental help.

## Data Availability

All the data are fully available without restriction at: Ahmad, Basaruddin (2021), “Farooq Ahmad Chaudhary 2020—psychosocial”, Mendeley Data, V1, https://doi.org/10.17632/kw4t627mmt.1 (https://data.mendeley.com/datasets/kw4t627mmt/1).

## Abbreviations

DMFT: Decayed, missing, filled teeth
OHI-S: Simplified oral hygiene index
CPI: Community periodontal index
OHIP: Oral health impact profile
HADS: Hospital anxiety and depression scale
SWAP: Satisfaction with appearance scale
BRS: Brief resilience scale
RSES: Rosenberg self-esteem scale
MSPSS: Multidimensional scale of perceived social support
